# Transcriptional Regulation on Aneuploid Chromosomes in Diverse *Candida albicans* Mutants

**DOI:** 10.1038/s41598-018-20106-9

**Published:** 2018-01-26

**Authors:** Christopher Tucker, Soumyaroop Bhattacharya, Hironao Wakabayashi, Stanislav Bellaousov, Anatoliy Kravets, Stephen L. Welle, Jason Myers, Jeffrey J. Hayes, Michael Bulger, Elena Rustchenko

**Affiliations:** 1 Department of Biochemistry and Biophysics, University of Rochester Medical Center, Rochester, New York USA; 2 Division of Neonatology and Program in Pediatrics Molecular and Personalized Medicine, Department of Pediatrics, University of Rochester Medical Center, Rochester, New York USA; 3 Department of Medicine and Center for Pediatric Biochemical Research, Department of Pediatrics, University of Rochester Medical Center, Rochester, New York USA; 4 University of Rochester Genomic Research Center, Rochester, New York USA; 5 Center for Pediatric Biochemical Research, Department of Pediatrics, University of Rochester Medical Center, Rochester, New York USA

**Keywords:** RNA, Transcriptomics, Transcriptomics

## Abstract

*Candida albicans* is a diploid fungus and a predominant opportunistic human pathogen. Notably, *C. albicans* employs reversible chromosomal aneuploidies as a means of survival in adverse environments. We previously characterized transcription on the monosomic chromosome 5 (Ch5) that arises with adaptation to growth on the toxic sugar sorbose in the mutant Sor125(55). We now extend this analysis to the trisomic hybrid Ch4/7 within Sor125(55) and a diverse group of three mutants harboring a single Ch5. We find a similar pattern of transcriptional changes on either type of aneuploid chromosome within these mutants wherein expression of many genes follows chromosome ploidy, consistent with a direct mechanism to regulate genes important for adaptation to growth. In contrast, a significant number of genes are expressed at the disomic level, implying distinct mechanisms compensating for gene dose on monosomic or trisomic chromosomes consistent with maintaining cell homeostasis. Finally, we find evidence for an additional mechanism that elevates expression of genes on normal disomic Ch4 and Ch7 in mutants to levels commensurate with that found on the trisomic Ch4/7b in Sor125(55). Several of these genes are similarly differentially regulated among mutants, suggesting they play key functions in either maintaining aneuploidy or adaptation to growth conditions.

## Introduction

*Candida albicans* is a unicellular fungus that lives as part of normal human gut or genital microflora, but is an important opportunistic infectious agent in immune-compromised individuals. The diploid genome of *C. albicans* is organized into 8 pairs of chromosomes that can exhibit instability resulting in rearranged chromosomes or aneuploidy, which is well-tolerated by the organism^1–4^. Moreover, in *C. albicans*, reversible loss or gain of one copy of a specific chromosome or large portions of chromosomes appears to provide a means for the organism to adapt and survive in unique adverse environments^3–6^. A well-studied example of such control is a reversible loss of one copy of chromosome 5 (Ch5), which confers laboratory resistance to caspofungin (Cas^R^), an important antifungal from the echinocandin class^6^, and also allows for the utilization of the otherwise toxic sugar L-sorbose (Sou^+^)^4,7^. Sorbose is known to kill fungi in a manner similar to echinocandins (reviwed in Yang *et al*.)^6^. It was proposed that one Ch5 is lost because Ch5 carries multiple genes for negative control of the Cas^R^ and/or Sou^+^ phenotypes^8,9^. Although the detrimental consequences of aneuploidy due to gene imbalances are well described, recent studies with *Saccharomyces cerevisiae* demonstrated that aneuploidy can confer a fitness advantage under adverse conditions^10,11^.

We have previously addressed the intriguing question of how aneuploidy affects transcription in *C. albicans*. For this purpose, we analyzed the transcriptional profile of the monosomic Ch5 in the mutant Sor125(55), a.k.a. Sor55, which has lost one Ch5 as an adaptation to growth on sorbose as a sole source of carbon. We found a complex pattern of transcriptional regulation in which many genes are expressed at half the level seen in disomic strains, as would be expected, but many other genes are expressed at or near the same level as seen in disomic strains^12^. We proposed that aneuploidy in *C. albicans* is coupled with transcriptional compensation for changes in gene dosage to maintain cellular homeostasis. Our study of Ch5 in the mutant Sor125(55) raised several questions that we wished to address experimentally. Does monosomic Ch5 in other, independently derived mutants also exhibit dosage compensation, and if so, in a similar pattern? Does transcription from a trisomic chromosome, which we identified in Sor125(55), also involve dosage compensation?

To address these questions, we have examined three additional mutants that have lost one copy of Ch5 in order to adapt to growth in the presence of either sorbose or caspofungin. In addition, we examined transcription from the trisomic chimeric chromosome 4/7b of Sor125(55)^2^. Application of high stringency analysis to a diverse set of mutants shows underlying similarities in expression changes. In particular, while expression of the majority of genes on the monosomic Ch5 in the newly analyzed mutants were changed according to the ploidy change, as expected, a substantial number of genes are upregulated to the disomic level so as to compensate for gene dose. Moreover, likewise we find that the majority of genes on the Ch4/7b are upregulated coordinately with ploidy, but significant compensation to the disomic level also occurs. Finally, we also report an unknown mechanism in cells adapted to stress conditions that elevates gene expression on normal disomic Ch4 and Ch7 to the levels observed in mutant cells harboring trisomic Ch4/7b.

## Results

### General approach and verification of chromosome condition

Our earlier analysis of the Sou^+^ mutant Sor125(55), indicated that Ch5 monosomy, responsible for adaptation to sorbose, directly results in a two-fold down-regulation of many Ch5 genes, as expected, while a substantial subset of Ch5 genes (at least 30%) remains expressed at the diploid level, presumably due to transcriptional compensation for gene dose^12^. Somewhat surprisingly, we also found that a small number of Ch5 genes are excessively upregulated, despite the loss of DNA. In order to determine if these observations are a general feature of related aneuploidy in C. albicans, we analyzed two additional mutants carrying a single Ch5: JMC200-3-4 and SMC60-2-5, which were selected for laboratory resistance to caspofungin, and a third mutant, Sor1210(60), a.k.a. Sor60, which was selected for resistance to sorbose. Each mutant was derived from a different genetic background (Table 1). The data for Sor125(55) were re-analyzed here using a more rigorous approach. In addition, we recently reported that both Sor125(55) and its parental strain, 3153A, harbor hybrid chromosomes resulting from a reciprocal exchange between one Ch4 and one Ch7 of the reference strain SC5314, which we term Ch4/7a and Ch4/7b^2^. In Sor125(55), Ch4/7b is duplicated, so genes on this hybrid chromosome, together with the corresponding portions of the intact Ch4 and Ch7, have a copy number of three^2^. Therefore, we also analyzed the transcriptional profile of “trisomic” Ch4/7b in Sor125(55). Notably, the Sor1210(60) mutant is also derived from the 3153A parental strain, and harbors Ch4/7a and Ch4/7b; however, Ch4/7b has not been duplicated in this mutant^2^.

**Table 1:** *C. albicans* strains used in this study.

Strain	Description	Phenotype	Source
SC5314	Reference sequencing strain, normal diploid	Cas^S^ Sou^−a^	A.D. Johnson laboratory
SMC60-2-5	Same as SC5314, but Ch5 monosomy, *MTL***a**. Caspofungin-generated	Cas^R^ Sou^+^	ref.^6^
JRCT1	Clinical isolate, normal diploid	Cas^S^ Sou^−^	ref.^6^
JMC200-3-4	Same as JRCT1, but Ch5 monosomy, *MTLα*. Caspofungin-generated	Cas^R^ Sou^+^	ref.^6^
3153 A	Laboratory strain, normal diploid	Cas^S^ Sou^−^	ref.^23^
Sor125(55) a.k.a. Sor55	Same as 3153 A, but Ch5 monosomy, *MTLα*, and Ch4/7b trisomy. Sorbose-generated	Cas^S^ Sou^+^	refs^2,6,12^
Sor1210(60) a.k.a. Sor60	Same as 3153 A, but Ch5 monosomy, *MTL****a***. Sorbose-generated	Cas^R^ Sou^+^	refs^6,24^

^a^The majority of *C. albicans* strains do not utilize sorbose. However, monosomy of Ch5 confers sorbose utilization or the Sou^+^ phenotype.

Caspofungin susceptibility of JMC200-3-4 or SMC60-2-5 was determined with spot assay and with standard broth microdilution method^13^, while Sor1210(60) or Sor125(55) were spot assayed^6^. Note that Sor125(55) is more susceptible to caspofungin than its parental strain 3153 A^6^.

We verified the chromosomal condition of all mutants using pulsed field gel electrophoresis, as well as DNA-seq (JMC200-3-4 and SMC60-2-5) and Southern blot analysis [Sor125(55) and Sor1210(60)]^2,12,13^.

### Gene expression analyses

We analyzed gene expression genome-wide in the three additional mutants described above by RNA-seq (Materials and Methods), to determine if a common subsets of genes were differentially regulated in mutants compared to parental strains (see Table 1) in association with monosomic Ch5. Analysis of RNA-seq data using CuffDiff2 (at FDR < 0.05) identified 392, 21, and 394 genes as differentially expressed in JMC200-3-4, SMC60-2-5, and Sor1210(60), respectively (Table 2), as compared to their parental strains. A total of five differentially expressed genes were in common across all three mutants (Table 2). Out of the five, three were on Ch5, with CHT2 (orf19.3895) and TRR1 (orf19.4290) downregulated, while expression of CAG1 (orf19.4015) was increased in the mutants. Two other genes, FAR1 (orf19.7105), found on Ch7, and CEK2 (orf19.460), on ChR, were both upregulated. Expression changes noted for CAG1, FAR1, and CEK2 that are involved in mating were as expected, as Ch5 monosomy carrying MTL (Mating Type Like) locus contributes to mating competence. Furthermore, downregulation of CHT2 was previously reported on the monosomic Ch5 in various strains^6,9^. Applying a similar stringent threshold on the expression array data for Sor125(55) which contains a monosomic Ch5 and a trisomic Ch4/7b did not identify any differentially expressed genes (Table 2). CHT2 and TRR1 were also downregulated and CAG1 was upregulated in Sor125(55), but these genes did not pass our stringent threshold of FDR < 0.05. No differences were detected with the aforementioned approach despite the fact that previous analyses documented significant differences in expression of a fraction of genes in Sor125(55) compared to its parental strain^12^. This result suggests that at this extreme level of stringency differences observed in gene expression in mutants vs parentals detailed above are at very high level of confidence.

**Table 2:** Number of differentially expressed genes and genes in common in mutants JMC200-3-4, SMC60-2-5, Sor1210(60) and Sor125(55).

Test	Threshold	Ch5 monosomy			Common in 3 Mutants	Ch5 monosomy Ch4/7b trisomy	Common in 4 Mutants
		JMC200-3-4	SMC60-2-5	Sor1210(60)		Sor125(55)*	
CuffDiff2	FDR < 0.05	392	21	394	5	0	0
CuffDiff2	p < 0.05	1257	577	1204	154	191	15

^*^Differential expression was assessed by *t* test.

As the stringent analysis employed above identified no differentially expressed genes in Sor125(55), we then further assessed expression differences at a reduced threshold by CuffDiff2 at unadjusted p < 0.05. As presented in Table 2, this approach revealed a total of 154 differentially expressed genes (112 downregulated and 42 upregulated) in common across the three mutants with a Ch5 monosomy only, as well as a total of 15 genes (13 downregulated and 2 upregulated) in common across all four mutants. By this approach, *TRR1* and *CAG1* were among the 15 genes common for all mutants.

We, then, used semi-quantitative RT PCR to validate expression changes of *CHT2*, *TRR1*, and *CAG1* in Sor125(55), as well as in two other representative mutants (Table 3). According to this method, *CHT2* and *TRR1* were consistently downregulated, while *CAG1* was upregulated in all mutants. These results are consistent with previously reported analyses of *CHT2* expression in SMC60-2-5, and Sor125(55)^6,13^, as well as for *CAG1* in Sor125(55) and Sor1210(60)^12^.

**Table 3:** Expression change^a^ of *CHT2*, *CAG1* or *TRR1* carried on the monosomic Ch5. vs normal disomic Ch5 of the corresponding parental strain, as determined with semi-quantitative RT-PCR from three independent RNA preparations^b^.

Gene	SMC60-2-5/SC5314	Sor1210(60)/3153A	Sor125(55)/3153A
*CHT2*	0.07 ± 0.03	0.63 ± 0.03	0.61 ± 0.07
*TRR1*	0.62 ± 0.09	0.63 ± 0.03	0.61 ± 0.07
*CAG1*	2.10 ± 0.15	2.39 ± 0.08	2.33 ± 0.08

^a^Expression change was determined as averaged ratio mutant/parent ± SD.

^b^The differences in expression between mutants and parentals, SC5314 or 3153A, were evaluated with Student’s *t* test showing all p-values < 0.05.

Based on CGD Gene Ontology Slim Mapper (Candida GO-slim Process), 9 out of 15 genes (60%) were identified as involved in regulation of biological processes, 6 (40%) are involved in filamentous growth, while 5 genes are involved in each of the following processes: response to chemical, transport, organelle organization, and conjugation.

**Figure 1:**
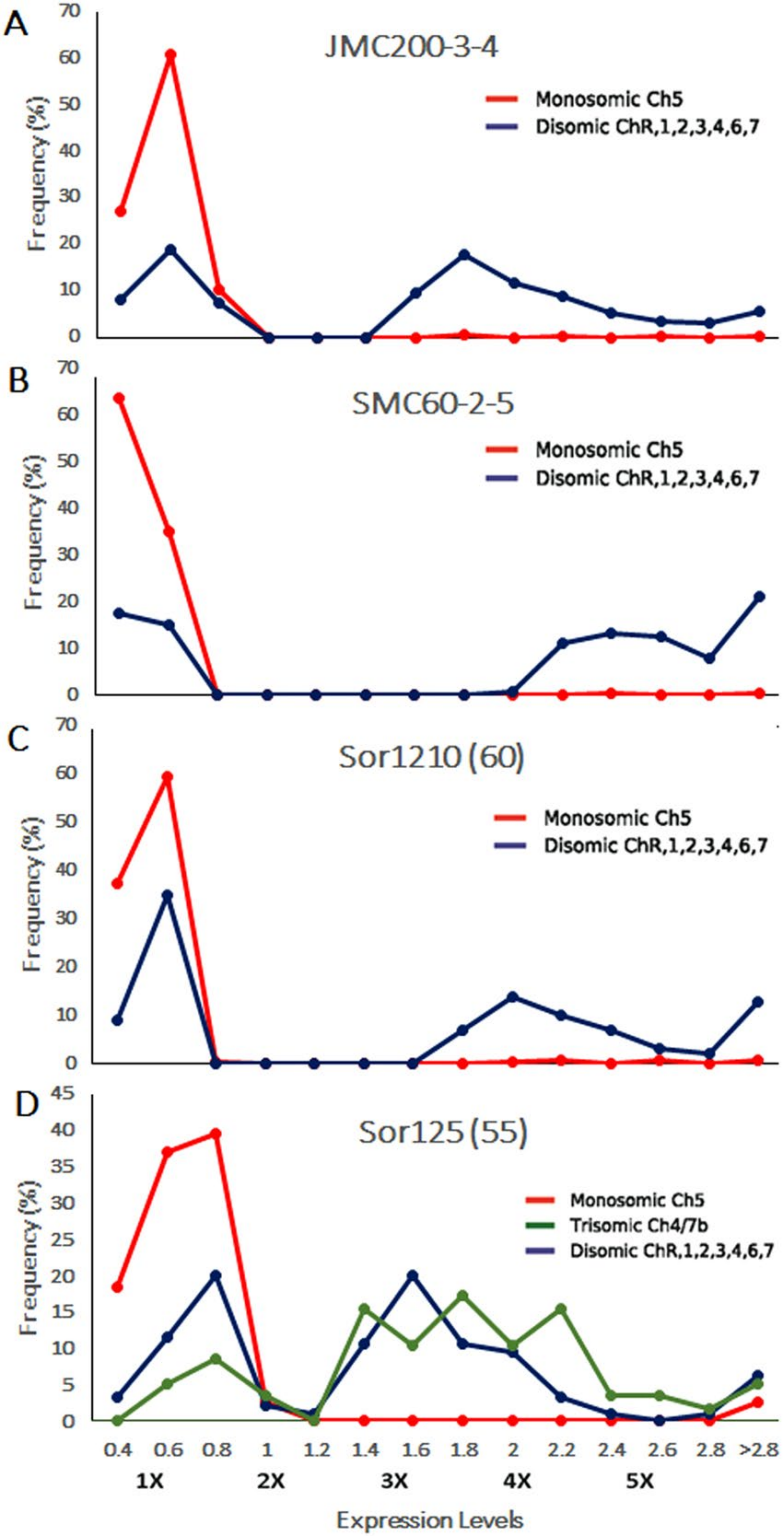
Distribution of differentially expressed genes present on monosomic Ch5 or trisomic Ch4/7b genes over expression levels. (**A**,**B**, and **C**) Distribution for monosomic Ch5 genes that were significantly different (CuffDiff2 at p < 0.05) in mutant strains [JMC200-3-4, SMC60-2-5, and Sor1210(60), as indicated]. (**D**) Distributions for monosomic Ch5 genes and the trisomic Ch4/7b genes that were significantly different (CuffDiff2 at p < 0.05) in Sor125(55). X-axis represents expression levels of the genes in mutants compared to parental strains and Y-axis represents the frequency of genes. Red lines represent genes on monosomic Ch5, blue lines represent genes on disomic chromosomes, and green line (in D) represents genes on the trisomic Ch4/7b. For RNA-seq analyzed mutants JMC200-3-4, SMC60-2-5, and Sor1210(60), for each of the significantly different gene, we derived the ratio of the level of expression averaged from three independent reads from each mutant strain compared to that observed in the corresponding parental strain. For expression array analyzed Sor125(55) we derived three independent sets of ratios for every mutant/parent pair, and plotted the average of the three bin sets (see also Materials and Methods). Note that genes exhibiting no significant expression changes are excluded from these graphs, including transcriptionally compensated genes on aneuploid chromosomes.

In order to present statistically significant (unadjusted p < 0.05) changes in gene expression in mutants compared to parental strains we plotted the fraction (%) of genes exhibiting similar changes in expression as a function of expression levels. Mutant/parental expression ratios of differentially expressed genes were grouped into bins spanning ranges from 0.4 to ≥2.8 and the proportions of genes falling within each bin were calculated. Expression ratios for genes from normal diploid chromosomes, monosomic Ch5, and the trisomic Ch4/7b are presented separately (Fig. 1). The binned levels of expression also represent multiples of the expected haploid expression level. Thus, for a normal, diploid chromosome, ratios of approximately 0.8, 1.0, and 1.2 (2×, see Fig. 1) cover the range of approximately equivalent gene expression between mutant and parent strains. However, for a monosomic chromosome, ratios of 0.8, 1.0, and 1.2 represent a 2-fold upregulation to the parental diploid level on a per-gene basis, as the expected ratio based on the haploid gene dose (and equivalent expression on a per-gene basis) would fall into the 0.4 to 0.6 bins (1×, see Fig. 1). For genes on the triploid Ch4/7b, ratios of 0.8, 1.0, and 1.2 would represent downregulation to the diploid level, while the expected triploid level would be 1.4 to 1.6 bins (3×, see Fig. 1).

For each mutant strain, ratios of mutant to parental gene expression for genes on disomic chromosomes exhibit both increases and decreases in expression genome wide (Fig. 1A–D, blue lines). A similar analysis of gene expression on monosomic Ch5 in the mutant strains JMC200-3-4, SMC60-2-5 and Sor1210(60) (Fig. 1A–C, red lines) shows a peak in the 0.4–0.6 range indicating that, in general, Ch5 monosomy results in proportionately lower expression of Ch5-linked genes in these mutants compared to their parental strains. In contrast, although Ch5 genes show an overall expression decrease in Sor125(55), the majority of genes exhibit ratios of 0.6–0.8, which approach the disomic level of expression (Fig. 1D, red line). This suggests, as previously reported^12^ that a robust and broad mechanism compensating for gene dose on the monosomic Ch5 is operative in the Sor125(55) mutant compared to the three new mutants strains harboring only Ch5 monosomy, which showed a much lower total fractions of apparently compensated genes. Note that the latter are not shown in Fig. 1, which shows only differentially expressed genes (but see below the sub-section “Genes on the monosomic Ch5 and trisomic Ch4/7b that are expressed at the disomic level”). Thus there is more similarity in expression changes among the mutants JMC200–3–4, SMC60–2–5 and Sor1210(60), which harbor only monosomic Ch5, but lack the trisomic Ch4/7b found in Sor125(55).

In addition, an examination of expression of genes present on the trisomic Ch4/7b in Sor125(55) showed the majority had expression ratios ranging from 1.4 to 2.2, indicating expression at trisomic or higher levels, as expected from gene dose. Remaining genes on Ch4/7b were expressed at monosomic or disomic levels.

### Gene expression analyses on the monosomic Ch5s and trisomic Ch4/7b

We next attempted to identify genes on chromosomes exhibiting aneuploidy by comparing expression of individual genes on monosomic Ch5s or trisomic Ch4/7b among the mutant strains. Application of CuffDiff2 (at FDR < 0.05) identified 125, 7, and 108 Ch5 genes as differentially expressed, respectively, for the mutants JMC200–3–4, SMC60-2-5, and Sor1210(60), which harbor only Ch5 monosomy (Table 4). As mentioned above for the global analysis, this very stringent approach did not reveal differentially expressed genes on monosomic Ch5 in Sor125(55).

**Table 4:** Number of differentially expressed genes and genes in common on the monosomic Ch5 in different mutants, JMC200-3-4, SMC60-2-5, Sor1210(60), and Sor125(55).

Test	Threshold	Ch5 Monosomy			Common in 3 Mutants	Ch5 monosomy Ch4/7b trisomy	Common in 4 Mutants
		JMC200-3-4	SMC60-2-5	Sor1210(60)		Sor125(55)*	
CuffDiff2	FDR < 0.05	125	7	108	3	0	0
CuffDiff2	p < 0.05	309	198	252	119	38	12

^*^Differential expression was assessed by Student’s *t* test.

Note that a total of 521 genes are present on Ch5 in the RNA-Seq data for JMC200-3-4, SMC60-2-5 and Sor1210(60) and 499 genes are present on Ch5 in the expression array data for Sor125(55).

This analysis identified three Ch5 genes that were similarly regulated among three mutants harboring only Ch5 monosomy, with downregulation of *TRR1* (~4 fold) and *CHT2* (~5 fold), as well as ~10–15 fold upregulation of *CAG1*, consistent with previous results (see “Gene expression analyses”). Even though these three genes did not meet the statistical threshold of being identified as differentially expressed in Sor125(55) from the expression array data, they show similar changes in magnitude and direction, and differential regulation was in fact subsequently validated by RT-PCR (Table 3).

We also identified differentially expressed genes by looking at all genes that were significantly different at a p-value <0.05. As presented in Table 4, this approach revealed a total of 119 genes differentially expressed in common across the three mutants harboring a single Ch5, out of a total of 521 Ch5 genes (23%). A total of 12 genes (3%) were identified with similar expression patters in common across all four mutants (including Sor125(55)), with 11 genes downregulated, including *TRR1* and *CHT2*, and one, *CAG1*, consistently upregulated in all four mutants.

**Figure 2:**
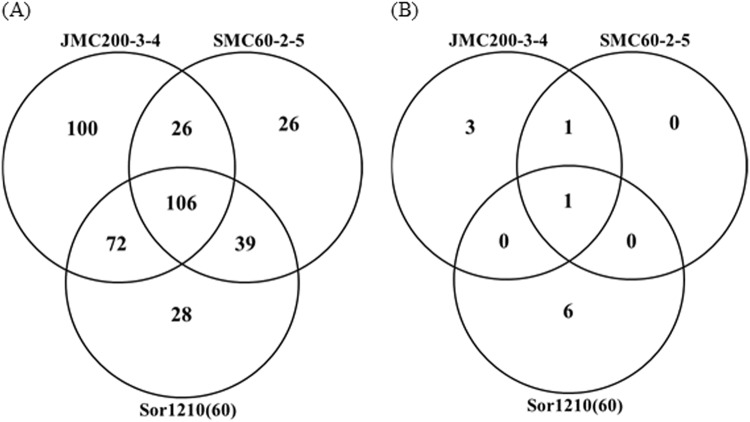
Venn diagrams showing 369 genes that were differentially expressed by CuffDiff2 at p < 0.05 on the monosomic Ch5 in a total of three caspofungin-tolerant mutants JMC200-3-4, SMC60-2-5, and Sor1210(60). Numbers in intersections of the circles represent the number of shared genes, while the number in the larger part of the circle represents the genes unique to that particular mutant. (**A**) Downregulated genes. (**B**) Upregulated genes.

When broken down by directionality of change, we found the highest overlap among downregulated genes. For example, 132 of a total of 369 (36%) Ch5 genes were downregulated in common between the mutants JMC200-3-4 and SMC60-2-5, which were derived from two different genetic backgrounds by exposure to caspofungin, and harbor monosomic Ch5 (Fig. 2A). Further, we found an overlap of 106 (27%) genes downregulated in common when the mutant Sor1210(60) was included in this comparison (Fig. 2A). Note that this latter mutant was derived from still another genetic background by exposure to sorbose but also harbors a monosomic Ch5. In contrast, we found very few upregulated Ch5-linked genes that were similarly differentially expressed across the mutants. Two genes, *CAG1* and *PGA37* (orf19.3923), are upregulated in both of the mutants generated by caspofungin exposure (JMC200-3-4 and SMC60-2-5) and only *CAG1* was also similarly upregulated in both the sorbose-generated mutants Sor1210(60) and Sor125(55) (Fig. 2B).

Of the 106 genes downregulated in common across the JMC200-3-4, SMC60-2-5 and Sor1210(60) mutants, the largest fractions play a role in the regulation of biological processes (32%), organelle organization (23%), and in RNA metabolic process (21%). Other functions include genes involved in response to stress (19%), filamentous growth (16%), cell-cycle (14%), transport (14%), response to chemicals (13%), and translation (12%), based on CGD Gene Ontology Slim Mapper (Candida GO-Slim: Process). Finally, 19% are involved in unknown biological processes.

Using CuffDiff2 (at p < 0.05) we identified a total of 51 genes differentially expressed out of 402 genes on the trisomic Ch4/7b. Almost all of the differentially expressed genes, 45 out of 51 or 90%, were upregulated (mutant to parent ratio of 1.4 or greater) consistent with the increased chromosome ploidy, while a small fraction of 6 genes, 10%, were downregulated (mutant to parent ratio of 0.8 or less).

As can be seen from Tables 2 and 4, inclusion of the Sor125(55) mutant consistently diminished the number of differentially expressed genes regulated in common between all mutants. This may be attributed to the fact that Sor125(55) not only harbors a monosomic Ch5 but, unlike the other mutants, has the additional aneuploid chromosome 4/7b and shows a much more robust transcriptional compensation of the monosomic (and trisomic) genes to the disomic level (Table 5). Interestingly, Sor125(55) is more susceptible to caspofungin than the parental strain (Table 1).

**Table 5:** Number of genes expressed at the disomic level on the monosomic Ch5 or the trisomic Ch4/7b in mutants JMC200-3-4, SMC60-2-5, Sor1210(60), and Sor125(55) and number of genes in common, as indicated.

	Ch5 monosomy			Common in 3 Mutants	Ch5 monosomy Ch4/7b trisomy	Common in 4 Mutants
	JMC200-3-4	SMC60-2-5	Sor1210(60)		Sor125(55)	
Ch5	49	71	85	3	151	1
Ch4/7b					102	

We also note that a substantial amount of the monosomic Ch5 or trisomic Ch4/7b genes in each mutant retained expression at the disomic level, i.e. were upregulated or downregulated, correspondingly, to compensate for gene dose. These genes are analyzed separately, below.

### PCR-based validation of expression changes on the trisomic Ch4/7b

We have previously validated the expression levels of many genes on the monosomic Ch5 in Sor125(55) using s.-q. RT PCR or Northern blot analyses^12^. Here we validated expression of four selected genes on the trisomic Ch4/7b that are expressed at either the trisomic or disomic levels, by s.-q. RT PCR: orf19.7096 and orf19.3833, that exhibit mutant/parent ratios of 1.1 (disomic level); and *GLN4* (orf19.7064), and *ERG26* (orf19.2909) that exhibit mutant/parent ratios of 1.5 (trisomic level) as determined by arrays (see Figure. S1 for an example of s.-q. RT PCR gel). We confirmed the expression ratios in three independent experiments. Orf19.7096 and orf19.3833 showed, respectively, averaged ratios of 0.95 and 0.97, while *GLN4* and *ERG26* showed, respectively, averaged ratios of 1.6 and 1.5, confirming results from the expression array determinations.

### Genes on the monosomic Ch5 and trisomic Ch4/7b that are expressed at the disomic level

**Figure 3:**
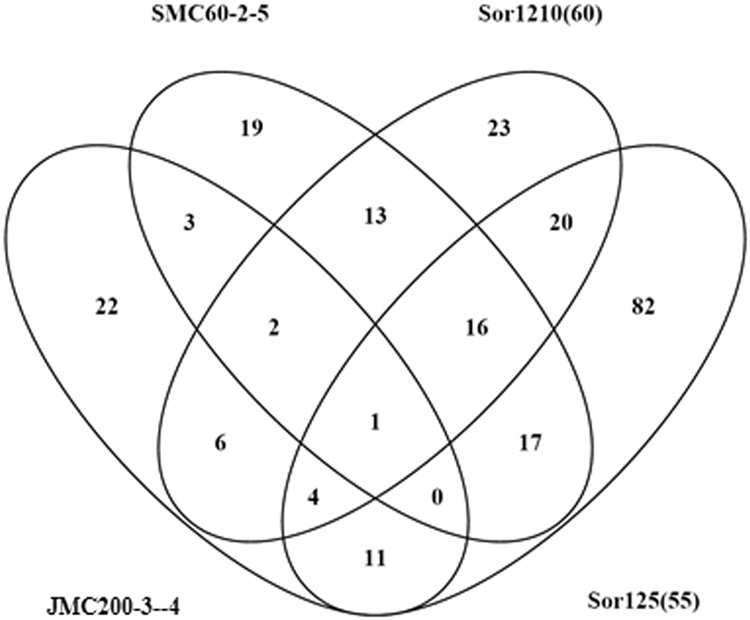
Venn diagrams showing genes that were expressed at disomic level on the monosomic Ch5 in all four mutants JMC200-3-4, SMC60-2-5, Sor1210(60), and Sor125(55). Numbers in intersections of the circles represent the number of shared genes, while the number in the larger part of the circle represents the genes unique to that particular mutant.

Genes expressed at the disomic level on the aneuploid chromosomes are of special interest, as this implies transcriptional compensation for the altered gene dose in aneuploid cells, presumably due to strict cellular requirements. We identified monosomic Ch5 or trisomic Ch4/7b genes in common among the mutants that are transcriptionally compensated to the diploid level of expression, which we took as mutant/parent gene ratios from 0.8 to 1.2. A total of 346 genes on monosomic Ch5 fit that criterion in at least one of the mutant strains: 49 (9.4%) in JMC200-3-4; 71 (13.6%) in SMC60-2-5; 85 (16.3%) in Sor1210(60); and 151 (31.5%) in Sor125(55) (Table 5). Pairwise comparisons of mutants revealed 6 genes regulated in common in both of the mutants generated by caspofungin exposure, JMC200-3-4 and SMC60-2-5; 13 genes in JMC200-3-4 and Sor1210(60); 16 genes in JMC200-3-4 and Sor125(55); 32 genes in SMC60-2-5 and Sor1210(60); 34 genes in SMC60-2-5 and Sor125 (55); and 41 genes in both of the mutants generated by sorbose exposure Sor1210(60) and Sor125(55) that are also of the same genetic background (Fig. 3).

Of six genes [*CUP1* (orf19.3940.1); *SNM1* (orf19.1927); *MES1* (orf19.3955); *SGE1* (orf19.1942); and uncharacterized orf19.577, and orf19.1121) shared between JMC200-3-4 and SMC60-2-5, three (*SGE1*, *CUP1*, and orf19.1121) were similarly expressed at diploid level in the Sor1210(60) (Table 5). Among those only orf19.1121 was also expressed at diploid level in the sorbose-generated Sor125(55) that, unlike other mutants, harbors both aneuploid Ch5 and Ch4/7b (Table 5). When compared across all four mutant strains, we identified only one gene in common expressed at the diploid level, among the ~500 genes present on Ch5 (p-value of 0.03 by hypergeometric test of overlap). Likewise, we find three genes similarly expressed at the disomic level across the three additional mutants (significant by hypergeometric test). Thus compensation of these genes appears to be significant for some biological process, and their role will be the subject of future studies.

A total of 102 (25%) out of 402 trisomic Ch4/7b genes, were downregulated to the disomic level, supporting the idea that dosage compensation in Sor125(55) is operative not only on the aneuploid chromosome of the type 2n−1 (Ch5), but also on the type 2n + 1 (Ch4/7b). (For the PCR-based validation of expression changes of the representative trisomic genes see above). Based on CGD Gene Ontology Slim Mapper (Candida GO-slim Process), 28% of genes were involved in organelle organization, 26% were involved in regulation of biological processes, 23% were involved in transport, while 26% were associated with unknown biological processes, among others.

### Comparison of genes expressed at the trisomic level on either normal Ch4 and Ch7 *or trisomic Ch4*/*7b*

Among the mutants we have analyzed, Sor125(55) is unique in possessing the hybrid duplicated Ch4/7b. We were therefore interested in the identities of Ch4- and Ch7-linked genes in mutants harboring normal, disomic Ch4 and Ch7 that exhibit upregulation to the trisomic level observed in Sor125(55), as it is possible that some of these genes may contribute to the formation of the monosomic Ch5. We first identified 131 genes on trisomic Ch4/7b in Sor125(55) that are expressed to the fully triploid or higher level of expression, defined as mutant/parent gene ratios of 1.4 or higher (Table 6). We then similarly identified genes in the mutants JMC200-3-4, Sor1210(60) and SMC60-2-5 that are upregulated to the trisomic or higher levels on disomic Ch4 or Ch7 [94 in JMC200-3-4, 108 in Sor1210(60) and 128 in SMC60-2-5], with a total of 12 (4%) genes [*orf19.3140.1, orf19.741, orf19.2905, RHD3* (*orf19.5305*)*, orf19.3108, ZRT1* (*orf19.3112*)*, orf19.3827, orf19.3806, HOS1* (*orf19.4411*)*, orf19.1247, RPT5* (*orf19.3123*)*, orf19.1277*] being regulated in common across all four mutant strains, suggesting that at least a subset of these genes are involved in accommodating the monosomic Ch5 condition (Table 6). Moreover, a total of 35 genes were upregulated in common between JMC200-3-4 and SMC60-2-5, while 45 genes were similarly in common between Sor1210(60) and Sor125(55).

**Table 6:** Number of genes expressed at the trisomic level or higher on the trisomic Ch4/7b in Sor125(55) and on the disomic Ch4 or Ch7 in JMC200-3-4, SMC60-2-5, and Sor1210(60).

	Ch5 Monosomy			Common in 3 Mutants	Ch5 monosomy Ch4/7b trisomy	Common in 4 Mutants
	JMC200-3-4	SMC60-2-5	Sor1210(60)		Sor125(55)	
Ch4/7b					131	
Ch4 & Ch7*	94	128	108	27		12

^*^Disomic genes that are expressed at the trisomic level or higher in JMC200-3-4, SMC60-2-5, and Sor1210(60) out of a total of 402 genes residing on the trisomic Ch4/7b.

To determine the significance of the 12 commonly upregulated genes, we performed a virtual experiment. Mutant/parent expression ratios were decoupled from the genes themselves and randomly assigned to the Ch4/7b-linked genes to generate shuffled datasets; this generated a new, random distribution that still mimicked the overall distributions observed in the mutant strains. We then determined the intersection of upregulated genes in 1 × 10e6 trials of these shuffled datasets. The simulation comparing four mutants produced median value of 4 with p-value = 0.029764 cutoff at 8. The 12 commonly upregulated genes gave a p-value of less than 0.01, well below the significance threshold of 0.05. This suggests that the number of upregulated genes we observed to be common among the mutant strains we have analyzed is highly significant, thus, indicating a specific mechanism for upregulation of those genes in different mutants.

### Expression of Ch5-linked genes previously identified as respon*ding to caspofungin exposure*

Ten Ch5-linked genes have been previously reported to change their expression after exposure to caspofungin^14,15^. These include ECM331 (orf19.4255) and ZCF14 (orf19.2647), that are induced, as well as SCW11 (orf19.3893), CHT2, FAS1 (orf19.979), FET33 (orf19.943), GIT3 (orf19.1979), MIG1 (orf19.4318), CCC2 (orf19.4328), and HAM1 (orf19.1108) (Table S2) that are repressed. We examined expression of these genes in the caspofungin-tolerant mutants JMC200-3-4, SMC60-2-5, and Sor1210(60) (Table 1). The Sor125(55) mutant, in contrast, exhibits increased susceptibility to caspofungin (Table 1), and so we also examined gene expression in this mutant in order to compare the expression of genes responding to caspofungin exposure between resistant and susceptible mutants.

Of the induced genes, in our expression profiles of caspofungin-tolerant mutants JMC200-3-4, SMC60-2-5, and Sor1210(60), *ECM331* is expressed at diploid or close to diploid level in two mutants and is decreased more than twofold in another mutant. Expression of *ZCF14* diminished twofold or more in all three mutants. It appears that the pattern of *ECM331* and *ZCF14* changes is rather mixed or inconsistent with literature requiring more genes of this class to be analyzed.

Of the 8 genes reported to be repressed in response to caspofungin, 6 showed downregulation in all three of our caspofungin-tolerant mutants. The remaining two genes were expressed at the disomic or close to the disomic level. Thus, overall, there was a strong tendency to downregulation. In Sor125(55), in contrast, those genes had mixed expression from more than two-fold downregulation to full or close to full compensation.

### Expression of essential genes

A computer search of the CGD revealed a total of 15 essential genes on Ch5 and 11 on Ch4/7b (Table S3). Inspection of the expression profiles among all mutant strains indicated that the essential genes on the monosomic Ch5 were predominantly downregulated with 40 of the 64 total cases expressed at monosomic expression levels or lower. In only 11 cases were essential genes upregulated to an intermediate level, mutant/parent ratio of 0.7–0.8, and in only 9 cases to the full diploid level (in 4 cases there was no data). This reflects that expression of the majority of essential genes on Ch5 tends to follow the ploidy of the chromosome. Of the 11 essential genes on the trisomic Ch4/7b in Sor125(55), 2 genes were expressed at the triploid level and 2 other genes at tetraploid level, whereas 4 genes were expressed at the diploid level and 2 genes expressed at an intermediate level between haploid and diploid levels (in 1 case there was no data). The data do not indicate that Ch4/7b essential genes are preferentially up- or downregulated in response to aneuploidy.

### Expression changes analyzed by chromosomal position on Ch5 and Ch4/7b

We asked whether changes in expression from a parental to a mutant strain are related to chromosomal position (Figure S2). We chose a representative mutant carrying a monosomic Ch5, JMC200-3-4, and grouped Ch5-linked genes in this mutant according to their mutant/parent expression ratios to define three classes: (1) genes with ratios consistent with change in ploidy (twofold downregulation); (2) genes with ratios indicating expression at the disomic level; and (3) genes exhibiting overexpression to the trisomic level and above. We similarly defined three classes of Ch4/7b-linked genes in the Sor125(55) strain: (1) genes with mutant/parent expression ratios consistent with change in ploidy (1.5-fold upregulation); (2) genes exhibiting expression at the disomic level; and (3) genes exhibiting downregulation to the monosomic level. In the graphical representations (Figure S2), clustering of genes with similar expression changes on aneuploid chromosomes is not evident. Similarly, no clustering of twofold or more upregulated or downregulated genes was previously found on the monosomic Ch5 in Sor125(55)^12^. The data therefore indicate that up- or down-regulation of gene regulation on a monosomic Ch5 is not regulated at a locus- or domain-wide level, but suggests instead a gene-by-gene mechanism.

### Evaluation of similarities in regulation on different monosomic Ch5s

In order to better illustrate the similarities/differences of the expression of genes on different Ch5s, we performed comparison of expression ratios mutant/parent for each Ch5 gene between pairs of mutants. Expression ratios of every gene in two mutants were plotted against each other on the abscissa or ordinate using gnuplot software (Figure S3A–F). We found, as expected, that most of clustering occurs for downregulated genes and there is more similarities in the pairwise comparisons of three new mutants, i.e., not involving Sor125(55) (Figure S3A–C). Those scatter plots also had higher correlation coefficient R^2^: 0.825; 0.533, and 0.590, respectively. However, despite lower correlation coefficients in pairwise comparisons involving Sor125(55) (Figure S3D–F), there was still much clustering of genes pointing to similarities in the control of genes on different monosomic Ch5s.

## Discussion

We have previously analyzed gene expression from a monosomic Ch5 in the *C. albicans* mutant Sor125(55) and found that the monosomy condition controls many genes directly by downregulating them approximately twofold, but small number of genes are excessively upregulated despite the loss of one copy of Ch5. Interestingly, approximately 30% of genes across this chromosome were upregulated to the disomic level suggesting a dosage compensation mechanism is operative in this strain that is required for continued growth^2^. In addition, evidence for a transcriptional dosage compensation on the monosomic Ch5 is supported by the finding of a significant increase in the acetylation of histone H4 exclusively on Ch5 of Sor125(55)^16^. Here, in order to determine if mechanisms of transcriptional control that operate alongside inducible aneuploidy in *C. albicans* are more general, we have used a high stringency threshold to analyze transcription from a hybrid Ch4/7b that has been duplicated in Sor125(55), resulting in trisomy of some Ch4- and Ch7-linked genes that occur on this hybrid chromosome^2^. We found that the same principles of transcriptional control that operate on the monosomic Ch5 also operate on the trisomic chromosome: many Ch4/7b genes became upregulated accordingly to the increased chromosome ploidy, while a significant fraction (25%) of genes became downregulated to the disomic level, thus, implying compensation for the gene dose. If genes expressed at the disomic level are truly compensated for the dose, the corresponding hypothetical mechanisms operating at Ch5 or Ch4/7b have to be distinct, working in an opposing fashion to upregulate or downregulate genes, respectively.

To further investigate regulation on aneuploid chromosomes in *C. albicans*, we have analyzed gene expression from three additional mutant strains harboring monosomic Ch5s that independently arose from different genetic backgrounds. Similarly to the aneuploid chromosomes of Sor125(55), the majority of genes on the new monosomic Ch5s were expressed commensurately with the decrease of gene dose, i.e., were downregulated, while a second group of genes (9.4% to 16.3%) were expressed at the disomic level and thus were controlled via a distinct mechanism. The fraction of genes expressed at the disomic level in the new three strains analyzed in the current work was somewhat less than the 31.5% of genes elevated to the disomic level in the previously analyzed strain Sor125(55)^12^, suggesting that a more generalized compensation may be operative in the latter. It is possible that the additional aneuploid Ch4/7b enhances dosage compensation on Ch5, as approximately half as many genes (16.3%) are expressed at the disomic level in Sor1210(60), which is derived from the same parental strain as Sor125(55), but does not harbor a duplication of Ch4/7b or any other aneuploidy.

As presented above, there is a relatively large number of Ch5 genes expressed at the disomic level in each of the individual mutant strains. However, only one gene, of unknown function, was found to be common among all mutants and only three genes were common in three new mutants when applying a very stringent analysis. Both these overlaps were significant by hypergeometric test of commonalities. When examined by pairwise comparisons for all combinations of mutants, the number of genes regulated in common was larger. We found six among the pair of caspofungin-derived mutants (JMC200-3-4 and SMC60-2-5), which have different genetic backgrounds and 41 for the pair of sorbose-derived mutants with the same genetic background [Sor1210(60) and Sor125(55)]. These analyses suggest a significant role of the genetic make-up and/or of the toxic substance to which mutants became adapted and that all mutants utilize a similar mechanism of transcriptional control associated with chromosome aneuploidy.

Among the three new mutants [JMC200-3-4, SMC60-2-5 and Sor1210(60)], which harbor only Ch5 monosomy, we observed a degree of similarity in expression changes, be it either for genes across all chromosomes (five differentially expressed genes in common), or only restricted for genes located on Ch5 (three differentially expressed genes in common). The results of the differential expression analysis from the RNA-seq data for these three mutants were not as closely matched to the Sor125(55) mutant, which may be attributed to multiple factors, including the difference in platform used for expression profiling. Sor125(55) expression profiling data was generated on microarrays which are limited to detecting transcripts that correspond to existing genomic sequence information, while RNA-seq is capable of interrogating both known transcripts and exploring new ones, and as such is better suited for discovery-based studies. In addition, RNA-seq has the ability to quantify a large dynamic range of expression levels, thereby enhancing the gene expression changes in the mutant strains. This may explain the fact that while we observed multiple genes showing consistent differential expression changes at high level of stringency in the three strains from RNA-seq data, we did not see changes in the microarray data at the same level of stringency.

We find that some genes on the normal disomic Ch4 or Ch7 in the three new mutants are upregulated to the trisomic and higher levels due to an unknown mechanism, and, importantly, 12 of those genes are upregulated in common among three new mutants and to levels commensurate to that within the Sor125(55) mutant harboring the trisomic Ch4/7b. An exciting possibility is that increased expression of these 12 genes is important for the formation and maintenance of the monosomic Ch5. It is unlikely that these 12 common genes are involved in adaptation to caspofungin, as Sor125(55) is more susceptible to caspofungin than its parental strain, unlike the three other mutants which exhibit decreased caspofungin susceptibility^6^. CGD Gene Ontology Slim Mapper analysis of these 12 genes did not identify any known biological processes or molecular functions, and hence identification of the relevant biological functions will require additional genetic studies.

In summary, various *C. albicans* mutants analyzed here showed similarities in transcriptional control on aneuploid chromosomes of either of two types, 2n + 1 (Ch4/7b) or 2n − 1 (Ch5). We show that a subset of genes on the monosomic Ch5 are similarly differentially regulated, suggesting key functions in either maintaining aneuploidy or adaptation to growth conditions. This is despite the mutants having different genetic backgrounds and being derived by adaptation to two different toxins, caspofungin or sorbose. We also obtained evidence that a substantial number of genes is expressed at the disomic level from aneuploid chromosomes, implying two distinct dosage compensation mechanisms operating on monosomic or trisomic chromosomes. We also obtained evidence for an additional mechanism operating in concert on normal disomic chromosomes that upregulates genes in the mutants. These hypothetical mechanisms and their role in establishing aneuploid states will be addressed in future studies.

## Materials and Methods

### Strains, media, and primers

Parental strains, their aneuploid mutants used in this study, as well as relevant phenotypes are described in Table 1.

Sorbitol medium was prepared by substituting glucose for 2% sorbitol in synthetic dextrose (SD) medium^17^. Cells were routinely grown at 37 °C.

Strains were stored at approximately −70 °C in 25% glycerol in order to interrupt cellular metabolism and to prevent induction and propagation of genetic instability. Care was taken to grow and handle strains with necessary precautions to prevent undesirable chromosome instability, as previously described^1,12,18^.

Genes and primers used in this study are listed in Table S1 in the Supplemental material.

### RNA-sequencing and analysis

For the determination of expression levels, we standardized the growth of C. albicans cells. Petri dishes with synthetic medium in which glucose was substituted for sorbitol were seeded with ~3000 colony forming units per plate and incubated until young colonies appeared that contained ~10^5^ cells/colony. Sorbitol plates were used to grow batches of independent cultures for each strain. Total RNA was prepared from three independent batches for each strain using the YeaStar^TM^ RNA kit (Zymo Research, Irvine, CA) per manufacturer’s recommendations. RNA concentration was determined with the NanoDrop 1000 spectrophotometer (NanoDrop, Wilmington, DE) and RNA quality assessed with the Agilent Bioanalyzer (Agilent, Santa Clara, CA). The TruSeq RNA Sample Preparation Kit V2 (Illumina, San Diego, CA) was used for next generation sequencing library construction per manufacturer’s protocols. Briefly, mRNA was purified from 100 ng total RNA obtained independently from the three replicates of each strain, with oligo-dT magnetic beads and fragmented. First-strand cDNA synthesis was performed with random hexamer priming followed by second-strand cDNA synthesis. End repair and 3′ adenylation was then performed on the double stranded cDNA. Illumina adaptors were ligated to both ends of the cDNA, purified by gel electrophoresis and amplified with PCR primers specific to the adaptor sequences to generate amplicons of approximately 200–500 bp in size. All together eighteen libraries were generated (three of parental, and three of mutants) for each strain comparison.

The amplified libraries were hybridized to the Illumina single end flow cell and amplified using the cBot (Illumina, San Diego, CA) at a concentration of 8 picomoles per lane. Raw reads generated from the Illumina HiSeq. 2500 sequencer were demultiplexed and an average of 18,433,500 single-end 100 bp reads were obtained per sample. Quality filtering and adapter removal was performed using Trimmomatic and trimmed/cleaned reads are then mapped with Short-read mapping package (SHRiMP)^19^. FPKMs and fragment counts were scaled using the median of the geometric means of fragment counts across all libraries, and differential expression of genes was assessed using CuffDiff2^20^ and corrected for multiple testing using False Discovery Rate (FDR) thresholding^21^.

Expression change was determined for each gene by ratio mutant/parent. For this, we averaged the gene expression values from three independent experiments with mutant and divided by the average of this gene expression in the respective parental strain from three repeats.

### Expression microarrays and analysis

We used custom-designed “CustomArray^TM^ 12 K” arrays from CombiMatrix Corp. (Mukilteo, WA) containing 11,898 unique probes that covered 6,327 ORFs found in assembly 19 of the Candida Genome Database (CGD) (http://www.candidagenome.org/)^22^. Probes were designed to correspond to 5′ and 3′ ends of each ORF. Expression arrays data were obtained from three independent experiments^12^. In this work, ORF annotations and chromosome positions were updated using genome assembly 21 of the CGD, resulting in a total of 6,001 genes.

Expression data were analyzed with R scripts, as described previously^12^. Genomewide differential expression was assessed using paired t test (two-tailed probabilities) in conjunction FDR based correction for multiple testing.

### RT-PCR analyses

Extraction of total RNA, RT-PCR, and s.-q RT-PCR analysis was performed as described previously^12^.

## Electronic supplementary material

Supplementary Information

## Data Availability

RNA-seq raw data are available at Short Read Archive (SRA), http://www.ncbi.nlm.nih.gov/sra, with the temporary submission ID SRP063348. Expression array raw data are available at Gene Expression Omnibus (http://www.ncbi.nlm.nih.gov/geo/query/acc.cgi?acc=) with the accession numbers GSM455127-GSM455132. Otherwise, all data generated or analyzed during this study are included in this published article (and its Supplementary Information files).
