# Author Correction: Transcriptional Regulation on Aneuploid Chromosomes in Diverse *Candida albicans* Mutants

**DOI:** 10.1038/s41598-019-44362-5

**Published:** 2019-07-05

**Authors:** Christopher Tucker, Soumyaroop Bhattacharya, Hironao Wakabayashi, Stanislav Bellaousov, Anatoliy Kravets, Stephen L. Welle, Jason Myers, Jeffrey J. Hayes, Michael Bulger, Elena Rustchenko

**Affiliations:** 1 Department of Biochemistry and Biophysics, University of Rochester Medical Center, Rochester, New York USA; 2 Division of Neonatology and Program in Pediatrics Molecular and Personalized Medicine, Department of Pediatrics, University of Rochester Medical Center, Rochester, New York USA; 3 Department of Medicine and Center for Pediatric Biochemical Research, Department of Pediatrics, University of Rochester Medical Center, Rochester, New York USA; 4 University of Rochester Genomic Research Center, Rochester, New York USA; 5 Center for Pediatric Biochemical Research, Department of Pediatrics, University of Rochester Medical Center, Rochester, New York USA

Correction to: *Scientific Reports* 10.1038/s41598-018-20106-9, published online 26 January 2018

The original version of this Article contained an error in the title of the paper, where the word “diverse” was incorrectly given as “divers”. This has now been corrected in the PDF and HTML versions of the Article, and in the accompanying Supplementary Information file.

