# Supplementary material for: Transcriptional Regulation on Aneuploid Chromosomes in Diverse Candida albicans Mutants
Source: Sci Rep. 2018 Jan 26;8:1630. doi: 10.1038/s41598-018-20106-9 (PMC5786073; doi:10.1038/s41598-018-20106-9)
Supplement: Supplementary file 1 — Supplementary Information [file 41598_2018_20106_MOESM1_ESM.pdf]

Supplementary Information

Transcriptional Regulation on Aneuploid Chromosomes in Diverse *Candida albicans* Mutants

Christopher Tucker, Soumyaroop Bhattacharya, Hironao Wakabayashi, Stanislav Bellaousov, Anatoliy Kravets, Stephen L. Welle, Jason Myers, Jeffrey J. Hayes, Michael Bulger, Elena Rustchenko

Table S1. Expression changes* of the Ch5 genes responding to caspofungin exposure

		Expression ratio mutant/parent			
**Gene**	**ORF**	**JMC200-3-4/ JRCT1**	**SMC60-2-5/ SC5314**	**Sor1210(60)/ 3153A**	**Sor125(55)/ 3153A**
ECM331[1]	19.4255	0.28	1.25	1.43	0.59
ZCF14[1]	19.2647	0.22	0.62	0.39	0.75
SCW11[2]	19.3893	0.10	0.32	0.33	0.50
CHT2[2]	19.3895	0.20	0.24	0.44	0.28
FAS1[2]	19.979	0.38	1.13	0.65	0.55
FET33[2]	19.943	0.55	0.62	0.52	0.82
GIT3[2]	19.1979	0.39	0.26	0.46	1.15
MIG1[2]	19.4318	0.77	0.30	0.43	0.67
CCC2[2]	19.4328	0.62	0.40	0.57	0.85
HAM1[2]	19.1108	0.71	0.95	0.46	0.79

* Expression changes determined as ratio mutant/parent.

Superscript 1 or 2 indicates, respectively, induction or repression due to cells' exposure to caspofungin, as found in literature.

Table S2. Expression changes* of the essential genes on Ch5 and Ch4/7b

			**JMC200-3-4/ JRCT1**	**SMC60-2-5/ SC5314**	**Sor1210(60)/ 3153A**	**Sor125(55)/ 3153A**
**ORF**	**Gene**	**Ch**	**Expression ratio mutant/parent**			
19.1934	*HST3*	5	0.51	0.56	0.44	0.74
19.1936	*SNF1*	5	0.64	0.57	0.76	0.7
19.1949	*VPS1*	5	0.41	0.9	1.1	0.92
19.1960	*CLN3*	5	NA	0.62	0.6	NA
19.3174	*CDC24*	5	0.35	0.41	0.38	0.61
19.3187	*ZNC1*	5	0.79	0.61	0.78	1.14
19.3190	*HAL9*	5	0.72	0.38	0.32	NA
19.3893	*SCW11*	5	0.1	0.32	0.33	0.50
19.4005	NA	5	0.61	0.99	0.88	0.94
19.4223	*GCD11*	5	0.54	0.77	0.63	0.73
19.4299	*MSW1*	5	0.51	0.43	0.53	0.67
19.4318	*MIG1*	5	0.77	0.3	0.43	0.63
19.567	*TFB3*	5	0.45	0.45	0.54	0.54
19.941	*SEC14*	5	0.51	0.47	0.43	0.47
19.976	*BRE1*	5	0.53	0.39	0.58	0.67
19.1261	*CGT1*	4/7b	0.98	1.24	1.15	1.44
19.2884	*CDC68*	4/7b	NA	1.38	1.57	0.92

19.2909	*ERG26*	4/7b	1.03	1.05	1.07	1.6
19.3087	*UBI3*	4/7b	0.83	0.88	0.86	1.1
19.3111	*PRA1*	4/7b	2.34	4.71	4.05	0.85
19.3780	NA	4/7b	0.54	0.91	0.60	NA
19.3794	*CSR1*	4/7b	NA	0.93	1.32	0.78
19.3796	NA	4/7b	1.41	0.73	1.12	1.02
19.3818	*GOA1*	4/7b	0.84	1.24	1.29	1.95
19.4410	*ALG1*	4/7b	1.30	0.80	1.01	2.0
19.7025	*MCM1*	4/7b	0.66	0.54	0.69	1.13

* Expression changes determined as mutant/parent expression ratio.

NA Not Available.

Table S3. List of genes and primers

**Gene**	**Primer**
orf19.7096	Ch7-7096F1; 5’-CAAGCTCAAAGACGGTCATGTGGA Ch7-7096R1;<br>5’-CAAGGTGGCCAAGAGATCGGAAA
GLN4<br>(orf19.7064)	Ch7- GLN4F1; 5’-AGAAGTGAAGGCTTCTCGTGGGTTG Ch7- GLN4R1;<br>5’-CGGTAGGCAACCAAATCCCACAT
orf19.3833	Ch4-3833F2; 5’-TGGAGCTCTGCTGCTCAATCCAA Ch4-3833R2;<br>5’-AGACGATCTTCATGGGCCACTCC
ERG26<br>(orf19.2909)	Ch4- ERG26F1; 5’-AAAGCCGCAGCTGAAGAAGCTGT Ch4- ERG26R1;<br>5’-TGAGCATCGGCTACATTTCCAACA
CHT2<br>(orf19.3895)	Ch5-F; 5’- TGCTCCACAATGTCCAATGT Ch5-R;<br>5’-AGGTTGGGCTAGGAGCTAGGA
TRR1<br>(orf19.4290)	Ch5-F; 5’-AACCCATTAGCTGTGATTGGTGGTG Ch5-R;<br>5’-AAATCTTGGTGGCTGGGATGTGAC
CAG1<br>(orf19.4015	Ch5-F; 5’-GGTGGGTTTACCCAACAGGAGAGA Ch5-R;<br>5’-TCTGTACCACCAGCAACATCAGCA
REX2<br>(orf19.1466)	Ch2-F; 5’-CAAGCTCAAAGACGGTCATGTGGA Ch2-R;<br>5’-CAAGGTGGCCAAGAGATCGGAAA

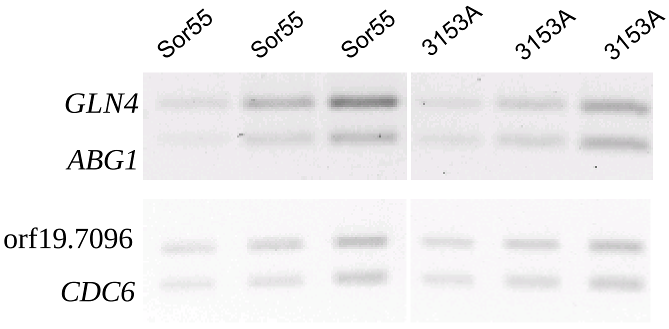

Figure S1. Analysis of RT-PCR products amplified from total RNA of strains 3153A and Sor125(55) using primers for GLN4 and orf19.7096. Those genes represent, correspondingly, increased and diploid level of expression on the trisomic Ch4/7b. Each gene was co-amplified with an indicated control. The following cycles are presented: GLN4, 27, 28, 29; orf19.7096, 26, 27, 28. The images of amplicons from at least three consecutive cycles in exponential phase were used for densitometry. Studied gene was normalized against the control gene by calculating mean ratio of densitometry values.

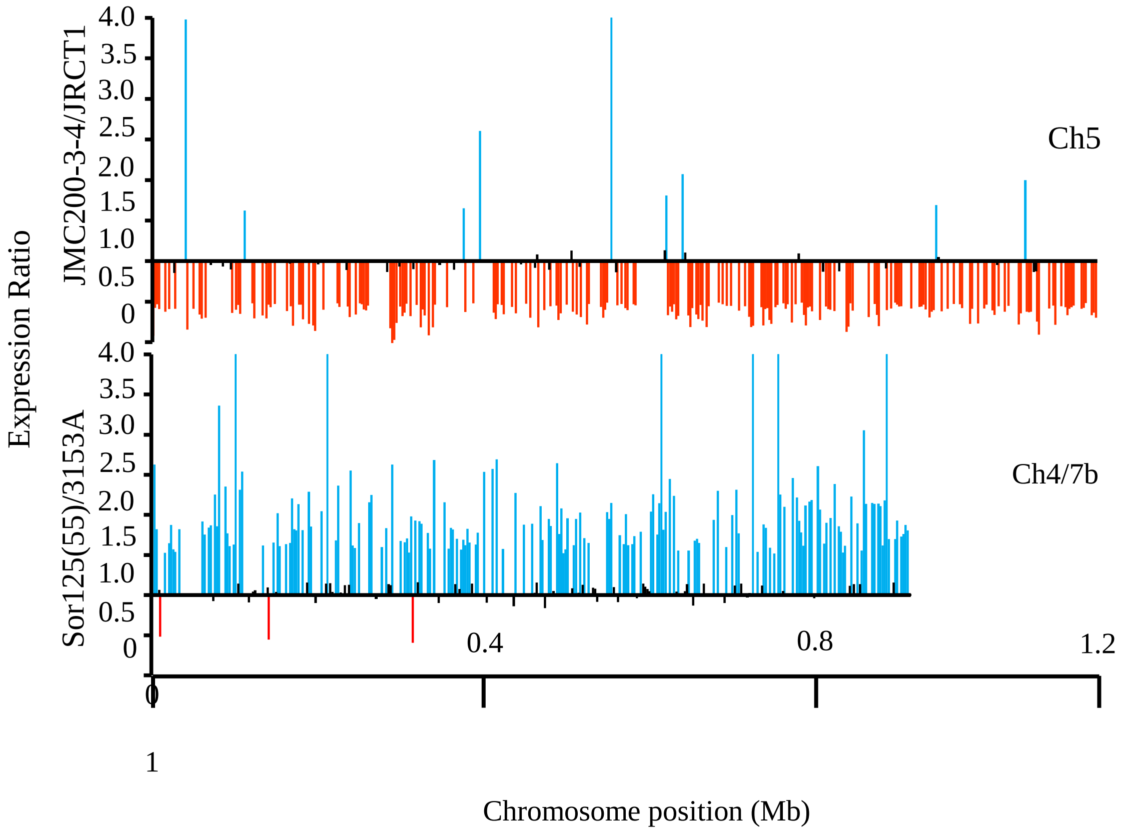

Figure S2. Distribution of selected expression ratios JMC200-3- 4/JRCT1 along the monosomic Ch5 and Sor125(55)/3153A along the trisomic Ch4/7b. Genes with expressions ratios ≤0.5 for a single chromosome expression level are in red, genes with expression ratios ≥1.5 for three chromosomes' expression level are in blue, and genes with expression ratios 0.9-1.1 for two chromosomes' expression level are in black. The expression of the upregulated genes is cut off at 4. ORFs are arranged in positions conform their coordinates on chromosome, as found in CGD.

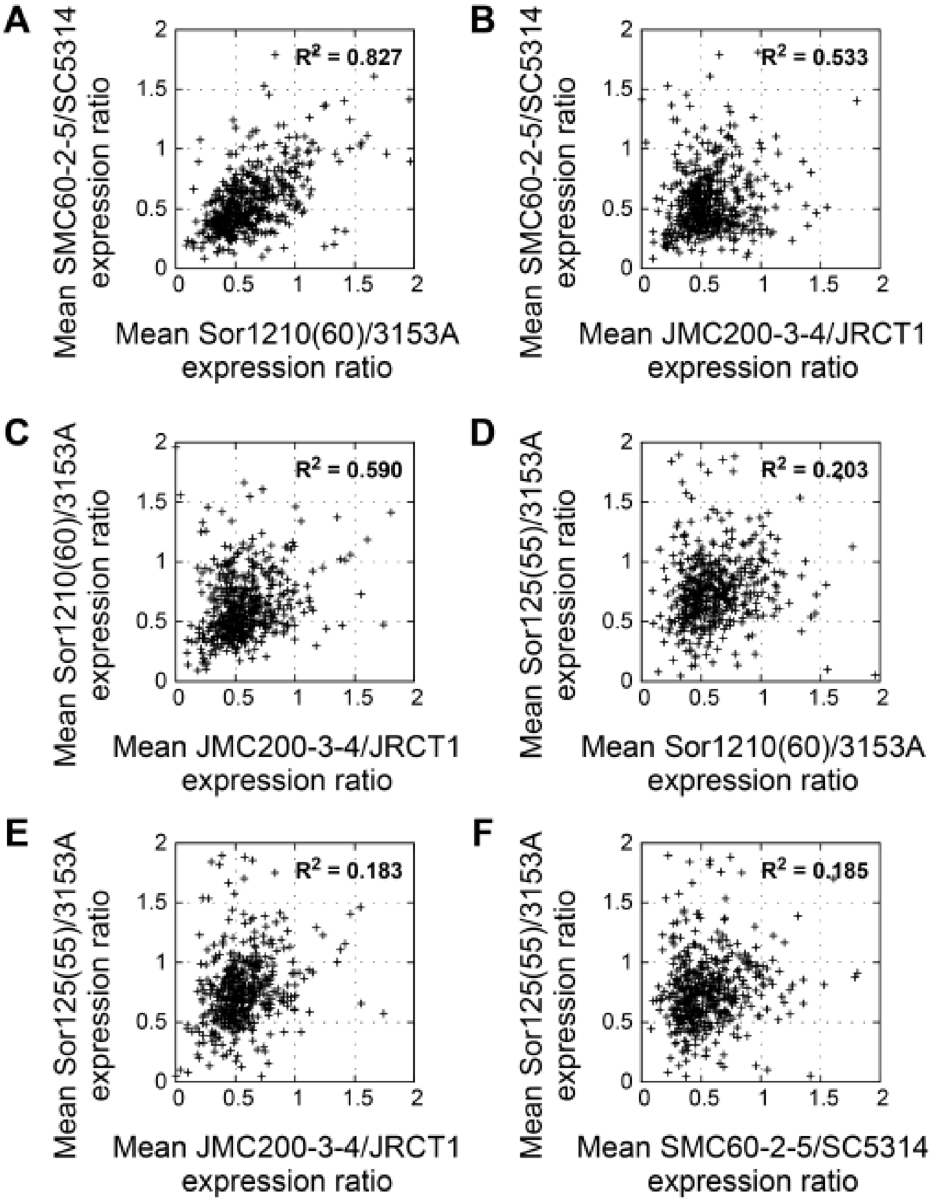

Figure S3. Combined plots of the expression of Ch5 genes in pairwise combinations of indicated mutants. The mean expression ratios mutant/parent for each Ch5 gene are plotted on the abscissa and on the ordinate. Every point on the plot corresponds to a gene. Correlation coefficient R^2 is shown for each ratio pair.
